# Integrative and comparative genomics analysis of early hepatocellular carcinoma differentiated from liver regeneration in young and old

**DOI:** 10.1186/1476-4598-9-146

**Published:** 2010-06-12

**Authors:** Dilek Colak, Muhammad A Chishti, Al-Bandary Al-Bakheet, Ahmed Al-Qahtani, Mohamed M Shoukri, Malcolm H Goyns, Pinar T Ozand, John Quackenbush, Ben H Park, Namik Kaya

**Affiliations:** 1 Department of Biostatistics, Epidemiology and Scientific Computing, King Faisal Specialist Hospital and Research Centre, Riyadh, Saudi Arabia; 2 Department of Medical Biochemistry/Obesity Research Center, King Saud University, Riyadh, Saudi Arabia; 3 Department of Genetics, King Faisal Specialist Hospital and Research Centre, Riyadh, Saudi Arabia; 4 Department of Biological and Medical Research, King Faisal Specialist Hospital and Research Centre, Riyadh, Saudi Arabia; 5 Liver Research Center, College of Medicine, King Saud University, Riyadh, Saudi Arabia; 6 Immorgene Concepts Ltd, Stockton-on-Tees, UK; 7 Duzen Laboratories, Istanbul, Turkey; 8 Department of Biostatistics and Computational Biology; Dana-Farber Cancer Institute, Boston, MA, USA; 9 The Sidney Kimmel Comprehensive Cancer Center at Johns Hopkins, Department of Oncology, Johns Hopkins University School of Medicine, Baltimore, MD, USA

## Abstract

**Background:**

Hepatocellular carcinoma (HCC) is the third-leading cause of cancer-related deaths worldwide. It is often diagnosed at an advanced stage, and hence typically has a poor prognosis. To identify distinct molecular mechanisms for early HCC we developed a rat model of liver regeneration post-hepatectomy, as well as liver cells undergoing malignant transformation and compared them to normal liver using a microarray approach. Subsequently, we performed cross-species comparative analysis coupled with copy number alterations (CNA) of independent early human HCC microarray studies to facilitate the identification of critical regulatory modules conserved across species.

**Results:**

We identified 35 signature genes conserved across species, and shared among different types of early human HCCs. Over 70% of signature genes were cancer-related, and more than 50% of the conserved genes were mapped to human genomic CNA regions. Functional annotation revealed genes already implicated in HCC, as well as novel genes which were not previously reported in liver tumors. A subset of differentially expressed genes was validated using quantitative RT-PCR. Concordance was also confirmed for a significant number of genes and pathways in five independent validation microarray datasets. Our results indicated alterations in a number of cancer related pathways, including p53, p38 MAPK, ERK/MAPK, PI3K/AKT, and TGF-β signaling pathways, and potential critical regulatory role of *MYC, ERBB2, HNF4A*, and *SMAD3 *for early HCC transformation.

**Conclusions:**

The integrative analysis of transcriptional deregulation, genomic CNA and comparative cross species analysis brings new insights into the molecular profile of early hepatoma formation. This approach may lead to robust biomarkers for the detection of early human HCC.

## Background

Hepatocellular carcinoma (HCC) is the fifth most common cancer type, and is the third leading cause of cancer mortality worldwide [1,2]. Recent reports show that HCC is becoming more wide-spread and has dramatically increased in North America Western Europe and Japan [2-4]. Additionally, there is an increasing incidence of the disease among younger age groups that warrants further investigation [5,6].

Recently considerable attention has been placed on global gene expression studies as well as genomic aberrations in order to understand the pathogenesis of HCC, and to look for possible early markers of detection [7-13]. Although notable successes have been achieved, there still exist significant challenges due to the heterogeneous nature of HCC (and other cancers) as well as the complexity of the molecular pathogenesis of this disease. Depending on etiological and accompanying pathological conditions, such as viral infection, cirrhosis, inflammation, fibrosis and others, the HCC signature genes identified thus far vary considerably. Additionally, the study of tumor formation in the liver is difficult due to the continuous transcriptome changes that occur during regeneration after hepatectomy [14], as well as age related gene expression changes [15-17]. Similarly, cancer progresses through a series of histopathological stages during which genetic alterations accumulate, and a natural consequence of this are the dynamic changes in gene expression patterns that occur during hepatocellular carcinogenesis. Developing animal models of HCC provide an experimental ground for dissecting the genetic and biological complexities of human cancer and contribute to our ability to identify and characterize pathogenic modifications relevant to early stages of cancer development and progression [18,19]. The previous studies have used cross-species comparative genomics approach successfully to understand the molecular pathogenesis of various cancers [20-23]. Hence, combining cross-species comparative and/or functional genomics approaches with independent datasets from human and animal models of HCC along with genomic DNA copy number alterations enhances the ability to identify robust predictive markers for HCC [23-26].

**Figure 1:**
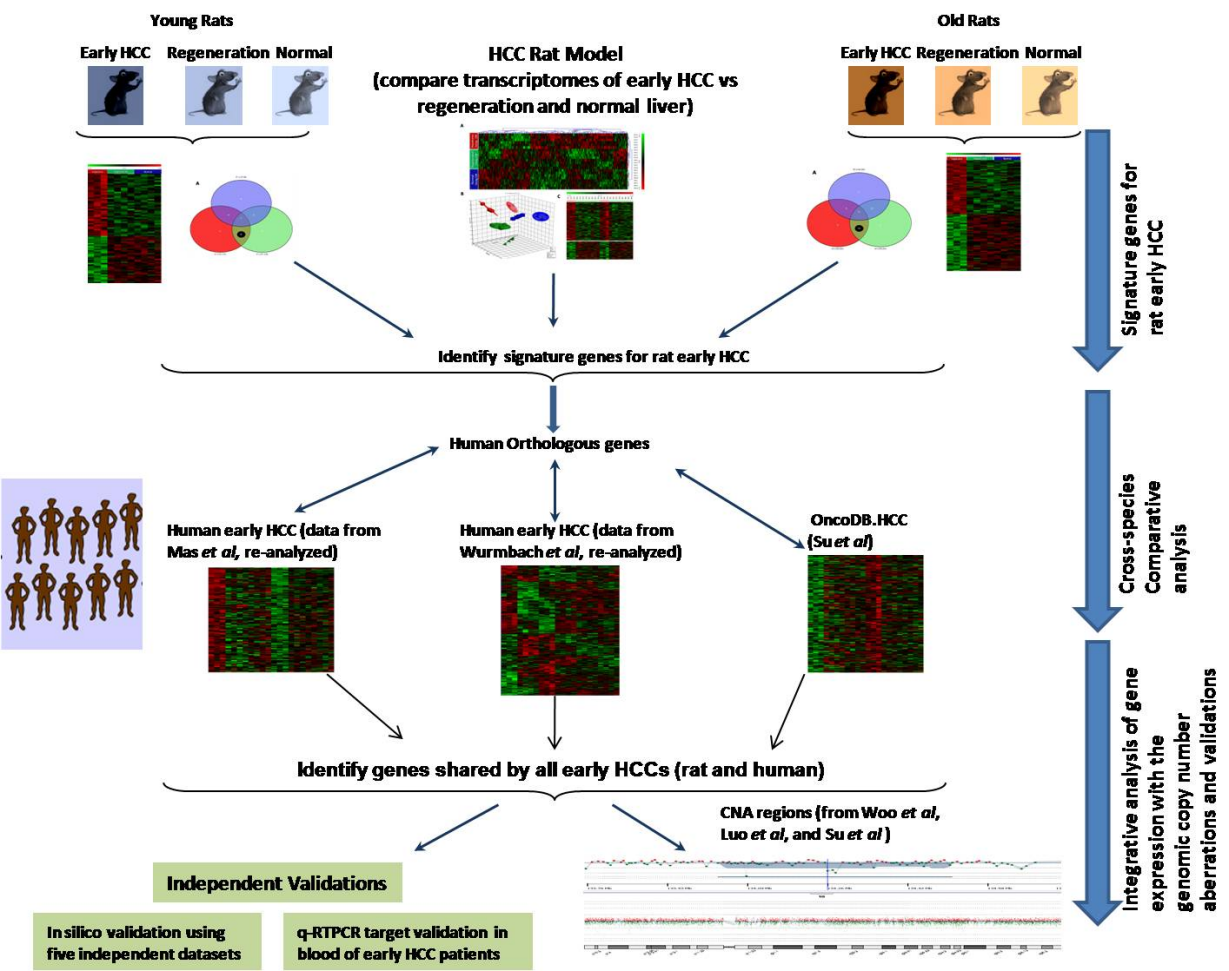
**Integrative and cross-species comparative genomics approach to identify evolutionary conserved inter-species biomarkers for early HCC differentiated from liver regeneration**. Gene expression signature for early rat HCC is differentiated from liver regeneration and normal liver in young and old using a microarray approach. Next, the cross-species comparative analysis was performed to identify genes that are conserved in early rat HCCs and in multiple independent early human HCCs, which would facilitate the identification of critical regulatory modules in the expression profiles. Finally, the integrative analysis of genomic copy number alteration (CNA) regions and gene expression profiles as well as independent validation analyses both in silico and with quantitative realtime RT-PCR were performed. HCC, hepatocellular carcinoma.

Here we present a comparative and integrative functional genomics approach to find an early marker for HCC. We developed a rat model and analyzed the transcriptomes of early HCC versus regenerated liver and normal liver in both young and old age animals using a microarray of more than 27,000 annotated genes from Celera and public repositories. We then performed cross-species comparative genomics analysis to identify genes that are conserved in rat and human early HCCs by re-analyzing independent datasets for human early HCC microarray expression profiling data [8,27] and also comparing with the Stanford HCC microarray data [7]. Finally, we performed an integrative analysis of DNA genomic copy number alterations (CNAs) and gene expression profiles (schematically outlined in Figure 1). Our findings include some genes already reported to be associated with human HCC, thus validating our approach. We also report many other novel genes which were not reported previously in liver cancers. Furthermore, we validated the high expression of eight potential biomarker genes from the blood of patients with early HCC using realtime RT-PCR. Our comparative and integrative genomics approach involving the integration of multiple high dimensional independent datasets may lead to robust biomarkers for the detection of early HCC.

## Results

### Gene Expression Profiling Confirms Pathological Classification

**Figure 2:**
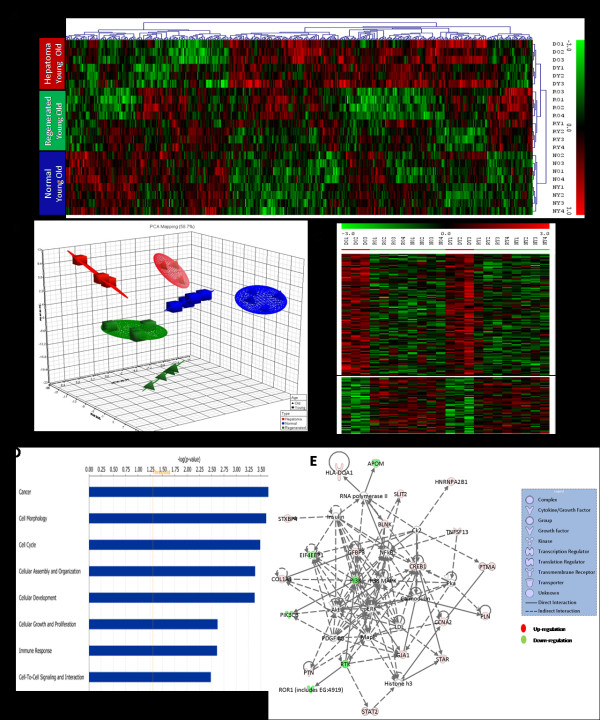
**Early HCC signature genes conserved across old and young rats**. (A) The unsupervised two-dimensional hierarchical clustering using genes that were significantly modulated due to treatment type across all samples (p < 0.01) clustered samples based on their treatment groups (HCC, regenerated and normal). Highly expressed genes are indicated in red, intermediate in black and weakly expressed in green. (B)The three dominant PCA components that contained around 60% of the variance in the data matrix separated samples based on treatment as well as age groups. (C) Heatmap of HCC signature genes conserved across old and young (D) Functional analysis of HCC specific genes. X-axis indicates the significance (-log p-value) of the functional association that is dependent on the number of genes in a class as well as biologic relevance (E) Gene interaction network of HCC specific genes generated by IPA analysis. Nodes represent genes, with their shape representing the functional class of the gene product, and edges indicate biological relationship between the nodes.

We performed genome-wide gene expression profiling of 24 samples for early HCC, regenerated liver and normal liver of both young and old rats using Applied Biosystems Rat Genome Survey microarray which includes more than 27,000 annotated genes from Celera and public repositories. To find genes that were differentially expressed across three different "treatment" types (i.e. early HCC, regenerated, and normal), and two age groups (old and young), we performed two-factor ANOVA to look for variations due to treatment, age and their interactions. The ANOVA identified 432 and 4063 genes that were significantly modulated by treatment type and age with p < 0.01, respectively. In addition, we found 322 genes that showed a significant interaction of age and treatment effect (data not shown). The unsupervised two-dimensional hierarchical clustering as well as principal component analysis (PCA) using genes which varied significantly with the treatment effect clustered samples according to their treatment type for both old and young (Figure 2A and 2B), hence supporting the conclusion that gene expression profiles robustly reflected the histological classification.

### Identifying HCC Specific Genes Conserved Across Old and Young

The ANOVA identified 432 genes that showed significant expression differences due to treatment in both age groups (p-value < 0.01) were subjected to a template matching algorithm (TMA) [28] to identify HCC specific genes conserved across both age groups. We identified 96 up-regulated and 38 down-regulated genes specific to HCC (template-match p-values < 0.01) (Figure 2C and Additional file 1).

The gene ontology and functional network analyses of HCC specific genes were performed using the Ingenuity knowledge base. The biological functions assigned to the dataset are ranked according to the significance of that biological function to the dataset. The enriched functional categories and diseases include carcinogenesis, cell cycle, immune response, cell morphology, cellular development, and growth and proliferation (Figure 2D). The PANTHER also revealed that signal transduction (p-value = 7.17 × 10^-4^), proteolysis (p-value = 1.59 × 10^-3^), cell motility (p-value = 3.91 × 10^-3^), immunity and defense (p-value = 1.07 × 10^-2^), and cell proliferation and differentiation (p-value = 4.9 × 10^-2^) were among the most enriched biological processes in the HCC specific genes. The most significantly altered pathways include p53, p38 MAPK, insulin/IGF pathway-protein kinase B signaling cascade, apoptosis, interleukin and integrin signaling pathways. The gene interaction network also corroborated with the altered pathways (Figure 2E).

### Age-Dependent Differences in Early HCC Differentiated from Regeneration

We found a significant interaction of age and treatment effect in our two-factor ANOVA analysis. Indeed, the hepatic transcriptome changes with ageing as in other cancer types, and age is a potential confounding factor embedded in gene expression profiles [15,16]. Therefore, we stratified samples as young and old cohorts, and identified HCC and regeneration specific genes using one-way ANOVA in each age group separately. The ANOVA identified 925 and 408 significantly dysregulated genes (up or down) due to the different treatment types in old and young animal groups (p-value < 0.02), respectively. The hierarchical clustering in both dimensions (samples and genes), as well as the PCA clearly separated samples based on the treatment type (Additional file 2), The gene expression clustering distance between the HCC group and the other two groups (regenerated and normal) was the greatest in both age groups (Additional file 2).

**Figure 3:**
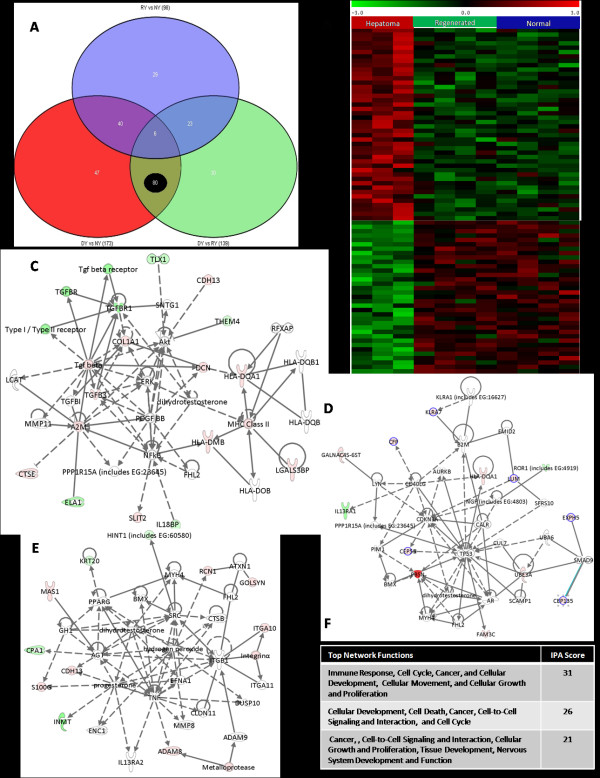
**Heatmap and gene interaction networks of early HCC specific genes in young**. (A) Venn diagram characterizing differential gene expression between and specific to different treatment types: early rat HCC (DY), regenerated (RY), and normal (NY). The number of HCC specific genes, 80, is circled in *black*. (B) Heatmap of HCC specific genes exclusively dysregulated (up/down regulated) in the HCC group only. (C-E) Top three scoring gene interaction networks (with highest relevance scores). Nodes represent genes, with their shape representing the functional class of the gene product, and edges indicate biological relationship between the nodes (*see legend *in Figure 2). (F) Top network functions associated with three networks shown. An IPA score of three indicates that there is 1/1000 (score = -log (p-value)) chance that the focus genes are assigned to a network randomly.

Early HCC signature genes in each age group were obtained by overlapping gene lists. Each circle in the Venn diagram represents the differential expression between two treatment types. We identified 80 genes and 100 genes specific to hepatoma in young and old, respectively (Figure 3A and Additional file 3). As seen from the heatmap of HCC specific genes, these sets of genes were exclusively up/down regulated in the HCC group only (Figure 3B and Additional file 3). The expression of *Pbsn, Lum, Adam8, Ctse, Calb3, Fbn1, Agtpbp1, Prom1, Ela1, Tnfsf13, *and *Ap2b1 *were significantly altered in both young and old rats with early HCC. The genes *A2m, Cdh13, Mas1, Slack, Cidea, *and *Dcn *were significantly dysregulated exclusively in HCC in the young; whereas *Cxcl5, Lox, Slc25a2, Rmt1, *and *Nid2, *were specific to HCC in old rats. We also identified regeneration specific genes in old and young rats in a similar approach (data not shown).

### Functional Comparison of Hepatoma and Regeneration in Young and Old

Early HCC signature genes in young animals were mainly associated with cancer, cell cycle, immune response, cellular function and maintenance, development, cell adhesion-mediated signaling, proteolysis, and signal transduction, whereas genes in old animals were highly associated with cancer, cellular movement, extracellular transport and import, cell adhesion, tissue development, cell morphology, cell-to-cell signaling and interaction. On the other hand, the regeneration specific genes were mainly associated with mRNA transcription and regulation, lipid metabolism, protein modification, protein phosphorylation, cell morphology, cellular development, small molecule biochemistry, and cellular growth and proliferation (Table 1). To gain more insights into HCC pathogenesis for young and old age early HCCs, we carried out gene interaction networks of HCC specific genes in young and old (Figure 3C, D and 3E and Additional file 3, respectively). The interaction networks highlight the important role of p53, p38 MAPK, ERK/MAPK, PI3K/AKT signaling, NF-κB and TGF-β pathways in early rat HCC.

**Table 1:** Functional comparison of hepatoma and regeneration in young and old

Molecular and Cellular Functions	Significance	Number of genes^1^	(%)
***Hepatoma in Young***			
Cancer	2.1 × 10^-3 ^- 4.9 × 10^-2^	12	33.3
Cellular Development	4.6 × 10^-3 ^- 4.9 × 10^-2^	8	22.2
Immune Response	2.5 × 10^-5 ^- 4.5 × 10^-2^	7	19.4
Skeletal and Muscular System Development and Function	9.1 × 10^-4 ^- 4.5 × 10^-2^	7	19.4
Cell Morphology	4.6 × 10^-3 ^- 4.9 × 10^-2^	7	19.4
Nervous System Development and Function	4.6 × 10^-3 ^- 4.9 × 10^-2^	6	16.7
Hair and Skin Development and Function	2.1 × 10^-5 ^- 1.4 × 10^-2^	4	11.1
Cell Cycle	8.6 × 10^-5 ^- 4.8 × 10^-2^	4	11.1
Cellular Function and Maintenance	2.1 × 10^-4 ^- 4.5 × 10^-2^	4	11.1
Amino Acid Metabolism	4.6 × 10^-3 ^- 4.9 × 10^-2^	3	8.3
Cell Death	4.6 × 10^-3 ^- 4.9 × 10^-2^	3	8.3
***Hepatoma in Old***			
Cancer	3.6 × 10^-3 ^- 4.8 × 10^-2^	15	46.9
Cellular Movement	2.3 × 10^-3 ^- 4.8 × 10^-2^	13	40.6
Cell-to-Cell Signaling and Interaction	4.9 × 10^-3 ^- 4.8 10^-2^	10	31.3
Tissue Development	4.7 × 10^-3 ^- 3.9 × 10^-2^	9	28.1
Cell Morphology	4.9 × 10^-3 ^- 4.9 × 10^-2^	9	28.1
Organ Development	3.1 × 10^-3 ^- 4.8 × 10^-2^	6	18.8
Embryonic Development	3.4 × 10^-3 ^- 4.7 × 10^-2^	5	15.6
Organ Morphology	3.1 × 10^-3 ^- 3.4 × 10^-2^	4	12.5
Cell Death	4.9 × 10^-3 ^- 3.9 × 10^-2^	4	12.5
Skeletal and Muscular System Development and Function	4.7 × 10^-3 ^- 1.9 × 10^-2^	3	9.4
Amino Acid Metabolism	4.9 × 10^-3 ^- 4.3 × 10^-2^	2	6.3
***Regenerated in Young***			
Cellular Growth and Proliferation	1.6 × 10^-3 ^- 4.8 × 10^-2^	5	38.5
Skeletal and Muscular System Development and Function	1.6 × 10^-3 ^- 3.7 × 10^-2^	5	38.5
Cell Morphology	1.6 × 10^-3 ^- 4.9 × 10^-2^	4	30.8
Cellular Assembly and Organization	1.6 × 10^-3 ^- 4.9 × 10^-2^	4	30.8
Cell-to-Cell Signaling and Interaction	1.6 × 10^-3 ^- 4.9 × 10^-2^	4	30.8
Small Molecule Biochemistry	1.6 × 10^-3 ^- 4.7 × 10^-2^	4	30.8
Tissue Development	1.9 × 10^-3 ^- 4.8 × 10^-2^	4	30.8
Cellular Development	1.6 × 10^-3 ^- 4.9 × 10^-2^	3	23.1
Cell cycle	1.4 × 10^-3 ^- 5.0 × 10^-2^	3	23.1
Amino Acid Metabolism	1.6 × 10^-3 ^- 4.6 × 10^-2^	2	15.4
Cellular Function and Maintenance	1.6 × 10^-3 ^- 3.2 × 10^-2^	2	15.4
Nervous System Development and Function	1.6 × 10^-3 ^- 4.8 × 10^-2^	2	15.4
***Regenerated in Old***			
Nervous System Development and Function	4.2 × 10^-5 ^- 4.2 × 10^-2^	22	37.9
Molecular Transport	3.8 × 10^-3 ^- 4.2 × 10^-2^	19	32.8
Cell Morphology	1.3 × 10^-4 ^- 3.8 × 10^-2^	15	25.9
Small Molecule Biochemistry	1.1 × 10^-3 ^- 4.2 × 10^-2^	15	25.9
Cellular Movement	1.5 × 10^-3 ^- 4.2 × 10^-2^	15	25.9
Cell Signaling	7.1 × 10^-3 ^- 2.3 × 10^-2^	15	25.9
Cell-to-Cell Signaling and Interaction	7.1 × 10^-3 ^- 3.9 × 10^-2^	12	20.7
Tissue Morphology	2.3 × 10^-4 ^- 4.2 × 10^-2^	11	19.0
Lipid Metabolism	1.1 × 10^-3 ^- 3.8 × 10^-2^	10	17.2
Cellular Growth and Proliferation	3.2 × 10^-3 ^- 3.7 × 10^-2^	10	17.2
Skeletal and Muscular System Development and Function	3.2 × 10^-3 ^- 3.7 × 10^-2^	9	15.5
Cellular Assembly and Organization	7.1 × 10^-3 ^- 4.1 × 10^-2^	8	13.8
Cellular Function and Maintenance	7.1 × 10^-3 ^- 3.5 × 10^-2^	7	12.1
Cellular Development	1.9 × 10^-4 ^- 3.7 × 10^-2^	5	8.6

^1^Thirty six, 32, 13, and 94 genes of the signature genes for HCC in young, HCC in old, Regenerated in young and Regenerated in old, respectively, mapped to corresponding genes in the knowledgebase.

### Cross-Species Comparative Genomic Analysis

**Figure 4:**
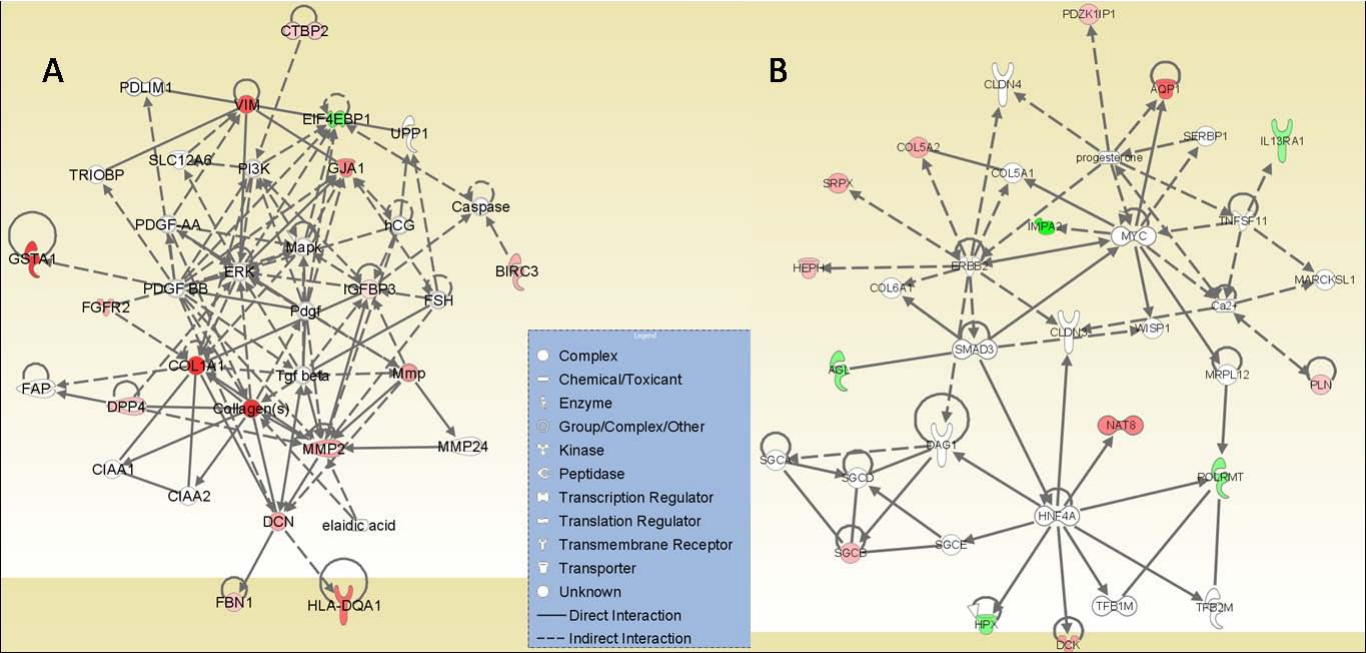
**The gene interaction networks of early HCC potential biomarker genes that are conserved in rat early HCCs and in multiple independent human early HCCs**. The network analysis of 35 early HCC signature genes indicated the activation of ERK/MAPK, PI3K/AKT and TGF-β signaling pathways, as well as potential critical regulatory roles of *MYC, ERbB-2, HNF4A*, and *SMAD3 *for early HCC; top two scoring networks are shown (A, B).

To identify how many of our early rat HCC signature genes were conserved in early human HCCs, we re-analyzed two independently performed microarray datasets for early human HCC from Wurmbach *et. al*. [8] and Mas *et. al*. [27]. The Wurmbach *et. al*.'s dataset composed of 19 early HCC patients and 10 normal controls, and the Mas *et. al*. dataset was composed of 16 cirrhotic livers with early HCC, 38 HCV associated HCC, and 19 normal livers. In addition, we also compared our signature genes with the OncodB.HCC database for 57 HCC patients from the Stanford HCC microarray data. The comparison of our rat early HCC signature genes (human orthologous) with the re-analyzed early human HCCs datasets revealed that 154 unique genes were conserved with early human HCCs (p < 0.001), and 35 of those were shared by all datasets analyzed (Table 2, Figure 1). We found that unsupervised clustering using the conserved signature gene list across species was sufficient to separate individuals in both Wurmbach's and Mas' human HCC samples as either early HCC patients or normal controls (data not shown). The gene interaction network analysis of the 154 signature genes indicated the importance of NF-κB, RAS and JNK activation in early hepatoma formation (Additional file 4). The network analysis of 35 cross-species conserved early HCC signature genes reveals the important roles of ERK/MAPK, PI3K/AKT, and TGF-β pathways (Figure 4). It also indicated a potential critical regulatory role of *MYC, ERBB2, HNF4A*, and *SMAD3 *for malignant transformation to early HCC (Figure 4B).

**Table 2:** List of 35 cross-species conserved early HCC signature genes with qRT-PCR and independent human/rat early HCC validations overlaid

*Gene Symbol^1^*	*Description*	*Chromosomal Loci*	*P-value*	*qRT-PCR*	*Independent Validation Datasets*.			
					
					*Human*		*Rat*	
					*Chiang et al.*	*Boyault et al.*	*Liao et al.*	*Liu et al.*
VIM*	vimentin	10p13	1.49E-16	DE^a^	NS	NS	NS	DE^c^
*DCN*	decorin	12q21.33	2.86E-15	DE^b^	DE^c^	DE^c^	DE^c^	DE^c^
EIF4EBP1*	eukaryotic translation initiation factor 4E binding protein 1	8p12	8.91E-15	NA	DE^c^	NS	NS	NS
*AQP1*	aquaporin 1 (channel-forming integral protein, 28 kDa)	7p14	1.15E-14	NA	NS	DE^d^	NS	NS
*IMPA2*	inositol(myo)-1(or 4)-monophosphatase 2	18p11.2	7.74E-14	NA	NS	DE^d^	NS	NS
*FBN1*	fibrillin 1	15q21.1	1.75E-12	NA	NS	NS	NS	NS
DCK*	deoxycytidine kinase	4q13.3-q21.1	4.22E-11	NA	DE^c^		DE^c^	NS
GJA1*	gap junction protein, alpha 1,43kDa (connexin 43)	6q21-q23.2	5.08E-11	DE^a^	NS	DE^c^	DE^d^	DE^c^
SP100*	SP100 nuclear antigen	2q37.1	6.95E-11	DE^a^	DE^c^	NS	NS	NS
SGCB*	sarcoglycan, beta (43kDa dystrophin-associated glycoprotein)	4q12	3.18E-10	NA	DE^c^	NS	NS	NS
FGFR2*	fibroblast growth factor receptor 2	10q26	1.15E-09	NA	NS	NS	DE^d^	NS
*ABCC9*	ATP-binding cassette, sub-family C (CFTR/MRP), member 9	12p12.1	1.75E-09	NA	DE^c^	NS	DE^c^	NS
MMP2*	matrix metallopeptidase 2	16q13-q21	1.22E-08	DE^a^	DE^d^	DE^c^	NS	NS
LGALS3BP*	lectin, galactoside-binding, soluble, 3 binding protein	17q25	4.29E-08	DE^a^	DE^d^	NS	NS	DE^c^
GSTA1*	glutathione S-transferase A1	6p12.1	5.16E-08	NA	NS	NS	NS	NS
*NAT8*	N-acetyltransferase 8	2p13.1-p12	3.20E-07	NA	DE^c^	DE^d^	DE^d^	NS
HMGB2*	high-mobility group box 2	4q31	3.34E-07	NA	DE^c^	DE^d^	DE^d^	NS
*EXPH5*	exophilin 5	11q22.3	6.15E-07	NA	DE^c^	NS	NS	NS
COL1A1*	collagen, type I, alpha 1	17q21.3-q22.1	7.62E-07	DE^a^	DE^d^	NS	NS	DE^c^
*POLRMT*	polymerase (RNA) mitochondrial (DNA directed)	19p13.3	7.95E-07	NA	DE^c^	NS	DE^c^	NS
*AGPAT2*	1-acylglycerol-3-phosphate O-acyltransferase 2 (lysophosphatidic acid acyltransferase, beta)	9q34.3	1.04E-06	NA	DE^c^	DE^c^	DE^d^	NS
CTBP2*	C-terminal binding protein 2	10q26.13	1.09E-06	NA	NS	NS	DE^d^	NS
*PROM1*	prominin 1	4p15.32	2.49E-06	NA	DE^c^	DE^d^	NS	NS
HPX*	hemopexin	11p15.5-p15.4	3.00E-06	NA	DE^c^	DE^c^	NS	NS
*HEPH*	hephaestin	Xq11-q12	3.15E-06	NA	NS		NS	DE^c^
HLA-DQA1*	major histocompatibility complex, class II, DQ alpha 1	6p21.3	2.68E-05	NA	DE^d^	DE^d^	NS	NS
*COL5A2*	collagen, type V, alpha 2	2q14-q32	3.88E-05	NA	DE^d^	NS	DE^d^	DE^c^
*BIRC3*	baculoviral IAP repeat-containing 3	11q22	6.19E-05	NA	DE^d^	NS	NS	NS
*DPP4*	dipeptidylpeptidase 4 (CD26, adenosine deaminase complexing protein 2)	2q24.3	0.00034	DE^a^	DE^c^	NS	DE^d^	NS
AGL*	amylo-1, 6-glucosidase, 4-alpha-glucanotransferase	1p21	0.000426	NA	DE^c^	DE^c^	DE^c^	NS
*PDZK1IP1*	PDZK1 interacting protein 1	1p33	0.001118	NA	DE^c^	DE^d^	NS	NS
*SRPX*	sushi-repeat-containing protein, X-linked	Xp21.1	0.001189	NA	DE^c^	DE^c^	DE^d^	NS
*IL13RA1*	interleukin 13 receptor, alpha 1	Xq24	0.004051	NA	DE^c^	NS	DE^c^	NS
*IGFBP3*	insulin-like growth factor binding protein 3	7p13-p12	0.006904	DE^a^	DE^c^	DE^c^	DE^c^	DE^c^
PLN*	phospholamban	6q22.1	0.028062	NA	NS	NS	NS	NS

^1 ^Genes with asterisk are also located in the chromosomal CNA regions.

NA indicates genes for which qRT-PCR data are not available.

^a^Validation was performed using whole blood of human early HCC patients (p < 0.05)

^b^Validation was performed on rat early HCC tissues (p < 0.05); five additional rat genes that are not listed in Table 2 are also confirmed (Figure 6).

^c^Differentially expressed (p < 0.02 and fold change (FC) > 1.8); NS: Not significant

^d^Differentially expressed: FC > 1.8, but not statistically significant (p > 0.05)

### Integrative Analysis of Transcriptional Deregulation with Genomic Copy Number Aberrations

**Figure 5:**
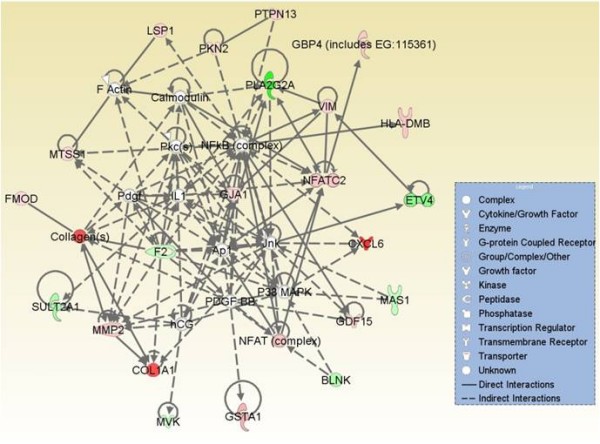
**The interaction network analysis of 75 early HCC signature genes conserved across species and having genomic alterations**. The network analysis of 75 cross-species conserved signature genes with CN alterations indicated the importance of NF-κB, p38 MAPK, AP1 and JNK activation in early hepatoma formation.

Various studies have reported chromosomal instability at chromosomal regions associated with many cancers, including human HCC copy number (CN) status [7,9,26,29,30]. These genomic modifications, which in part are reflected in changes in DNA copy number (CN), may alter the transcriptional control mechanism, and hence impact gene expression levels [31,32]. Hence, we compiled genes located in CNA regions reported in three independent genome copy number studies of human HCC [7,9,10]. The integration of 154 early HCC signature genes with the copy number data resulted in 75 genes that mapped to human CNA chromosomal loci genes including *COL1A1, CCNA2, NFATC2, F2, DCK, MMP2, GJA1, VIM, LGALS3BP, *and *SP100*. The interaction network of those genes further corroborated with the activation of the NF-κB, p38 MAPK, AP1, and JNK pathways (Figure 5). We found that almost 50% of the 35 cross-species conserved signature genes that are common to different types of early HCCs analyzed were mapped to genomic locations within the CNA regions (Table 2).

### Independent Validation Set Analysis

As a validation of our results, we analyzed four independently performed microarray datasets for early human and rat HCCs [19,33-35] using the analysis procedure defined in the "Methods" section on the new datasets. The first validation dataset was from Chiang *et.al*. [33] using Affymetrix short oligo arrays. The dataset was composed of 91 HCV-related HCC tumor samples, of which 65 were very early or early stage disease, which we used in our re-analysis and comparison. The re-analyzed validation dataset showed a significant number of genes (p < 10^-5^) in common with our analysis results. In fact, more than 50% of our cross-species conserved genes were also differentially expressed in early HCC compared to normal controls in the validation dataset (Table 2). The significance of overlaps was calculated using hypergeometric distributional assumption [36] and p-values were adjusted using Bonferroni correction for multiple comparisons [37]. In addition, unsupervised clustering was performed using our 35- gene signature to cluster the samples from Chiang *et. al*. We found that using our signature gene list was sufficient to separate individuals in Chiang *et. al*.'s study as either early HCC patients or normal controls (Additional file 5).

Moreover, we found a significant number of genes in common with the second dataset from Boyault *et. al*. [34] which consisted of 57 human HCCs and five samples of pooled non-tumorus tissues, and a third gene expression dataset from Liao *et. al*. [35] consisting of human HCC from various stages (we used expression data for only the early stage of the disease) (Table 2). Furthermore, we obtained consistent results with the DEN-induced HCC in rats from [19,38]. The IPA functional and network analysis of all validation datasets revealed a significant number of overrepresented functional categories and pathways in common with our results. Of note, cell death, cancer, cellular development, cellular growth and proliferation, organismal development, transport, and cell cycle came up as significantly enriched categories in both the validation datasets and our analyses. The interaction networks analyses of significantly dysregulated genes in validation datasets highlight the important roles of MYC, ERK/MAPK, AKT, NF-κB and TGF-β signaling pathways. The similar findings between our results and the independent validation sets argue against random chance accounting for the observed enrichment of these functional categories and pathways.

### Validation of Microarray Data for Early Rat HCC by Realtime RT-PCR

**Figure 6:**
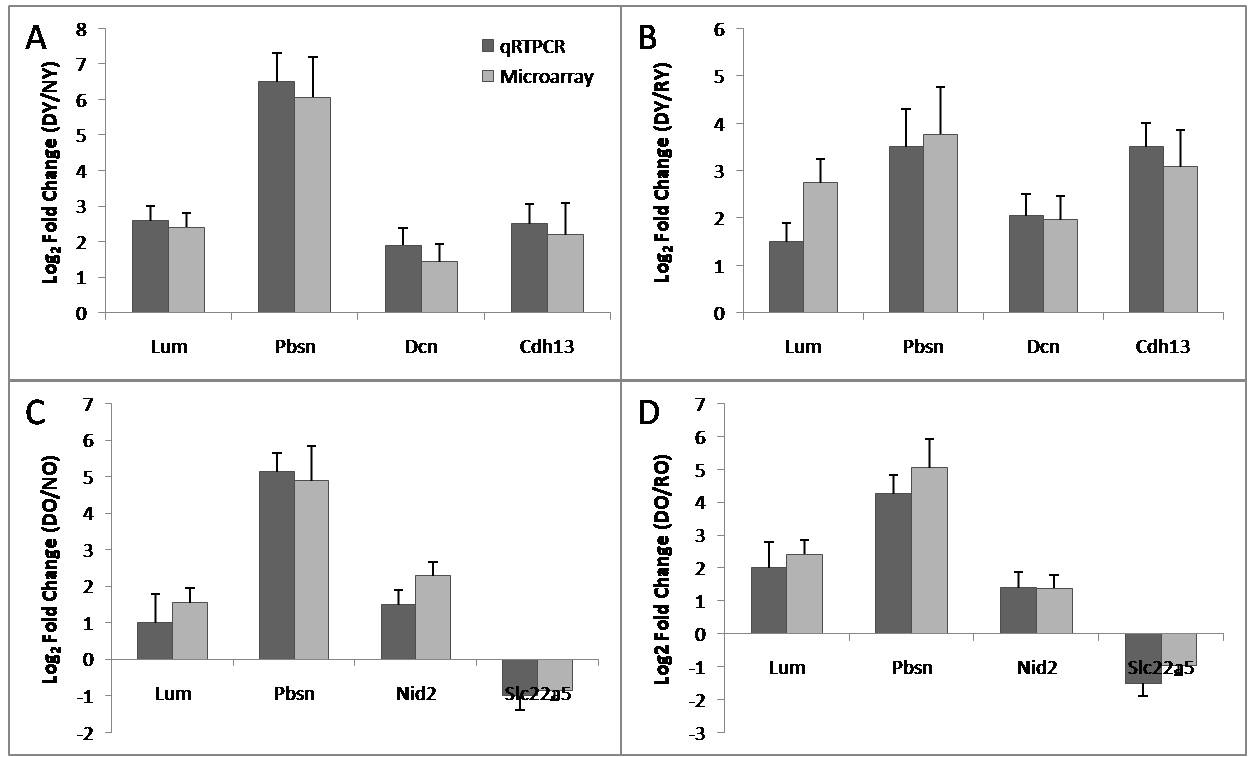
**Confirmation of the microarray gene expression for six randomly selected significantly regulated genes in rat early HCC by realtime qRT-PCR**. Ratio of expression (fold change) for each gene in (**A**) early HCC in young (DY) compared to normal (NY); (**B**) DY group to regenerated (RY), (**C**) early HCC in old (DO) compared to normal (NO); (**D**) DO group to regenerated (RO). A significant correlation existed between the microarray and realtime RT-PCR results (p < 0.001), thus demonstrating the reliability of our gene expression measurements. The fold changes were log_2 _transformed for both microarray data and real-time RT-PCR. Grey bars represent microarray hybridizations, and, and dark bars represent values from qRT-PCR. The error bar represents standard deviation (SD) over four experiments. P-values for triplicate analyses were all < 0.05.

To confirm the microarray results by an independent method, we validated expression levels of six randomly selected differentially regulated genes (*Pbsn, Cdh13, Lum, Nid2, Dcn, Slc22a5*) in early rat HCC by realtime quantitative RT-PCR. A highly significant correlation existed between the microarray and realtime RT-PCR results (r = 0.97, p value < 0.001) (Figure 6), thus demonstrating the reliability of our gene expression measurements. The selected genes and their interaction networks with other genes are shown (Figures 3C, D and 3E, 4A, and Additional file 3).

### Validation of Potential Biomarker Genes from Whole Blood of Patients with Early HCC Using qRT-PCR

**Figure 7:**
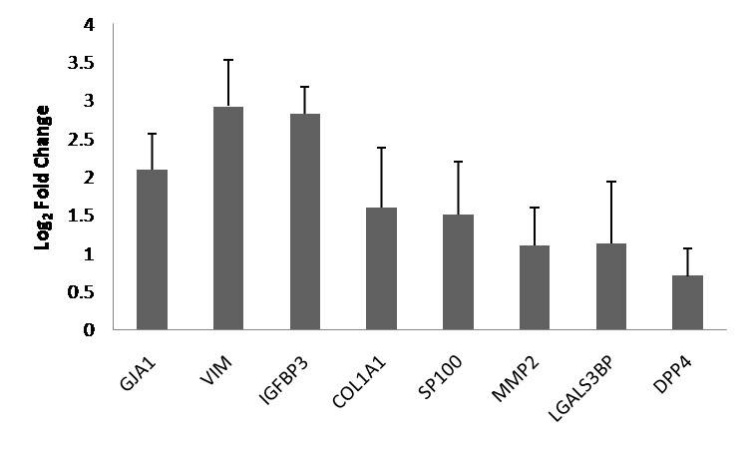
**Differential expression of a subset of genes was confirmed in whole blood of human early HCC subjects with qRT-PCR**. The up-regulation of expression of eight genes from Table 2 was confirmed in blood of early HCC patients compared to normal controls by using qRT-PCR. Values represent log_2 _of fold change in mRNAs in early HCC relative to the healthy control subjects (in every case, p < 0.05, Student's t-test). The error bar represents standard deviation (SD) over at least six experiments.

To further validate the differential expression of potential biomarker genes using realtime RT-PCR from the blood of early HCC patients and healthy control subjects (ten subjects in each group), we selected eight genes (*GJA1, VIM, IGFBP3, COL1A1, SP100, MMP2, LGALS3BP, *and *DPP4*) among early HCC gene signature with CNA (denoted with asterisk in Table 2) and were differentially regulated in at least one of the independent datasets. We confirmed a statistically significant increase in the expression of these biomarker genes in early HCC patients relative to healthy control subjects (p-value < 0.05) (Table 2, Figure 7); hence demonstrating the robustness of the cross-species integrated genomics procedure.

## Discussion

The present study sought to identify evolutionarily conserved inter-species biomarkers for early HCC differentiated from liver regeneration using integrative and cross-species comparative genomic approaches. The main contributions of this study are as follows: First, we developed a rat model of liver regeneration post-hepatectomy (return to quiescence), as well as liver cells undergoing malignant transformation and compared them to normal liver using a comprehensive microarray of 27,000 publicly available and Celera annotated rat genes. We included the liver regeneration in our model, as regeneration is a critical component in the surgical treatment of HCC, and frequently associated with HCC occurrence [39]. Though liver cells can regenerate, they do not typically transform and lead to HCC [40]. Therefore, an early HCC marker needs to be unique for the tumorigenic process and not overlap with the transcriptome changes that occur in regenerating or normal liver tissue. As ageing is also known to be a confounding factor embedded in gene expression profile data [15-17], we included age as a factor in our multi-factor statistical analysis, and identified age-specific differences in early HCC. Secondly, we performed cross-species comparative analysis to identify genes that are conserved in early rat HCCs and in multiple independently performed early human HCCs which would facilitate the identification of critical regulatory modules conserved across species in the expression profiles. Finally, we integrated genomic CNA data associated with the human HCCs with the transcriptomic profile, and performed validation analyses both in silico and with quantitative realtime RT-PCR (as schematically outlined in Figure 1). As CNAs have clear impact on expression levels in a variety of tumors [26,29,30], this dual strategy is very effective for interpreting the DNA and RNA level anomalies in cancer, in order to identify genes involved with tumor initiation and progression [24,26].

The validation analyses demonstrated great concordance of our results with other data sets using various microarray platforms. The ABI 1700 system has a unique approach in identifying dysregulated genes since it targets genes from both Celera and Public databases and utilizes chemiluminescently enhanced detection that is likely to determine relatively rare mRNAs. Also, our confirmatory quantitative realtime RT-PCR experiments displayed a strong correlation with the microarray results, adding to the validity of the present observations. This is in agreement with some recent studies showing a linear relationship for real-time and conventional reverse transcription and therefore validates the robustness of mRNA quantification using either microarrays or quantitative RT-PCR[41]. Hence this allowed us to identify potential biomarkers for human early HCC and to gain further insight into the mechanism of early hepatoma formation.

We performed a two-step algorithm to identify early rat HCC signature genes: In the first step, a two-way ANOVA was performed including treatment and age as well as their interactions into our statistical model and we identified genes uniquely expressed in early HCC in both young and old rats (Figure 2). In the second step, because the interaction between age and treatment was significant, we stratified our samples as young and old cohorts, and HCC specific genes were identified using two one-way ANOVA in each age group separately [42]. Finally, the HCC specific genes in both young and old identified in the two steps were combined before performing the cross-species comparison (as detailed in "Material and Methods" section, and schematically shown in Figure 1). When comparing the early HCC group with the regeneration or normal cohorts to identify the differentially expressed genes we used a set of criteria: S/N ratio > 3 in > 50% of the samples, a p-value < 0.02 and absolute fold change > 1.8. These observations are consistent with the study of Guo *et. al*., in which gene lists ranked by fold change and filtered with non-stringent statistically significant tests were more reproducible across platforms than those generated through other analytical procedures [43,44]. In addition, as Ghosh *et. al*. discuss on combining data from multiple gene expression studies, if two studies independently discover that the same gene/protein to be differentially expressed, then the chance of error is significantly reduced [45].

The comparison of our signature genes with three different independently performed early human HCC microarray data sets revealed a significant number of early rat HCC genes (human orthologous) conserved across early human HCCs (p < 0.001). Indeed, many of those genes were related to cancer. For example, *LUM, CCNA2, IGFBP3, HPX, COL1A1, SRPX, VIM, TGFBR1, DCN, MMP2, CD14, DCK, BIRC3, GJA1, LOX, SP100, PROM1 *and *CREB1 *were known to regulate tumorigenesis, neoplasia, apoptosis, growth, differentiation and proliferation. Some of the most significantly activated canonical pathways included hepatic fibrosis/hepatic stellate cell activation (*CD14, COL1A1, FGFR2, IGFBP3, MMP2*, and *TGFBR1)*, and p38 MAPK signaling pathways (*CREB1, PLA2G2A, TGFBR1*). The network analysis of early HCC signature genes indicated the activation of ERK/MAPK, PI3K/AKT, and TGF-β signaling pathway, and a potential critical regulatory role of *MYC, ERbB-2, HNF4A*, and *SMAD3 *for early HCC (Figures 2, 3 and 4). MAPKs are implicated in diverse cellular processes such as cell survival, differentiation, adhesion, and proliferation [46]. The gene network analysis of differentially expressed genes further confirmed the altered pathways. Moreover, it also indicated the importance of NF-κB, RAS and JNK activation in early hepatoma formation (Figure 5 and Additional file 4).The role of *MYC *in various types of carcinogenesis has been extensively investigated [47]. Most recently, JNK1 activation[48], and increased expression of ErbB-2 were found to be associated with HCC [49]. Thus, our current findings are consistent with previously performed independent cancer studies, including those for HCC. However, the novelty of our approach is that using comparative and integrative genomics, we provide evidence for the potential central role of these genes in the earliest phase of liver malignant transformation.

Our comparative genomics analysis resulted in a 35-gene cross-species conserved signature for all types of early HCCs. Over 70% of the conserved genes were associated with cancer according to the IPA knowledgebase, including *LGALS3BP*[50,51], *VIM*[52,53], *DCN*[54,55], *IGFBP3*[56], *FGFR2*[57], *GJA1*[58], *SP100*[59], *DPP4*[60], *PROM1*[61], *BIRC3*[62], *MMP2*[63], and *COL1A1*[64,65] (Table 2). Furthermore, using literature mining tools, such as MILANO [66], we found that almost 90% of our signature genes were reported to be cancer related.

There are areas of genomic instability reported in many cancers, including HCC, and some regions commonly exhibit either deletion or increased gene dosage, leading to changes in DNA copy number (CN) [9,26,29,30]. Integrating the gene expression with the CN data reveals the chromosomal regions with concordantly altered genomic and transcriptional status in tumors [24,32,67]. Hence, focusing on differentially expressed genes with concomitant altered DNA copy number may identify novel early HCC markers of malignant transformation and progression. The presence of altered DNA CN and LOH may contribute to cancer formation [30,31,68]. Therefore, the pattern of genomic modifications in a tumor represents a structural fingerprint that may include the transcriptional control mechanisms and locally impact gene expression levels [31,32]. We identified that more than 50% of our cross-species conserved early HCC signature genes were found to be copy number dependent (Table 2).

We found significant expression of *LGALS3BP *(Lectin, galactoside binding soluble 3 binding protein) and *COL1A1 *located on Chromosome 17q. The *LGALS3BP *is a 90-kD protein, designated serum protein 90 K that was found at elevated concentrations in the serum of patients with various types of breast, lung, colorectal, ovarian, and endometrial cancer [50,51]. It is a secreted glycoprotein that binds galectins, beta1-integrins, collagens, and fibronectin, and has some relevance in cell-cell and cell-extracellular matrix adhesion [69]. Another gene which could be a potential biomarker for early HCC is dipeptidyl peptidase IV (DPP4). DPP4 is a serine protease, which plays an important role in immune regulation, signal transduction, and apoptosis. It has been shown that DPP4 may have a critical function in tumor progression in several human malignancies [60,70]. Matrix metalloproteinases (MMP) also are involved with early carcinogenic events, tumor growth, tumor invasion and metastasis [63,71,72]. Matrix metalloproteinases (MMPs) are zinc-dependent endopeptidases that cleave and degrade a wide spectrum of extracellular matrix components, and are involved with extracellular matrix remodeling during the process of tumor invasion and metastasis [72]. Alterations in MMP expression and their endogenous inhibitor (TIMP) may contribute to HCC metastasis [71-73].

It is worth mentioning that, the gene "Probasin" (*Pbsn*), was significantly up-regulated (fold change > 15 in both old and young early rat HCCs). The high expression of *Pbsn *in our rat model was also confirmed with realtime RT-PCR. *Pbsn *is a member of the lipocalin family and has not yet been associated with HCC in rats and has no known human ortholog. However, it has been shown that *Pbsn *is highly expressed in prostate and implicated in both benign prostatic hyperplasia and prostate cancer [74-76] and taste bud tumorigenesis in rats [74]. Also since the promoter of this gene exhibits strong androgen receptor-specific and tissue-specific regulation, *Pbsn *is proposed to be a potential candidate for targeted therapies for advanced prostate cancer [77].

We have also found significant expression of lumican (*LUM*) and decorin (*DCN) *in both early rat and human HCCs. *LUM *and *DCN *are members of a small leucine-rich proteoglycan (SLRP) family. Lumican has been shown to participate in the maintenance of tissue homeostasis and modulation of cellular functions including cell proliferation, migration, adhesion, and differentiation [78]. Decorin has been reported to have a number of functions including suppressing cancer cell growth and metastasis andacting with extracellular matrix molecules to influence cell adhesion and fibril stability [55]. *DCN *acts as a natural inhibitor of TGF and is considered to be a specific antagonist of EGFR [54]. In addition, the altered expression of lumican and decorin has been associated with various human cancers including breast, pancreatic, lung, ovarian, melanoma, colorectal, osteosarcoma and ductal adenocarcinoma [54,78-82].

Genes whose protein products are released into the extracellular space would be ideal tumor markers for clinical applications, as it would be possible to detect these proteins in patients' biological fluids rather than through the use of invasive biopsies. Moreover, previous studies have found that cells derived from peripheral blood could be used to assess disease-associated gene signatures [83-89]. In our study, we confirmed the high expression of eight selected candidate biomarker genes (*GJA1, VIM, IGFBP3, COL1A1, SP100, MMP2, LGALS3BP, *and *DPP4) *by using realtime RT-PCR from the blood of early HCC patients. These genes and other potential biomarker genes identified through our integrated-comparative genomics approach (listed in Table 2) and their encoded proteins will be further studied in a large cohort of patients to determine if they have a role in early HCC pathology and if they could be novel early HCC biomarkers detectable in biological fluids.

## Conclusions

In summary, to our knowledge, this is the first study to examine HCC differentiated from regeneration in both old and young rats, and coupled with a cross-species comparative and integrative genomics approach to identify genes that could be potential biomarkers for early human HCC. The results of our study include the depiction of refined and delineated biological pathways differentially modulated in HCC that is built around TP53, p38 MAPK, ERK/MAPK, PI3K/Akt, NF-κB, TGF-β, MYC, and ERbB-2, including their target genes that were not previously implicated with early HCC. Our cross-species comparative and integrative genomics approach which involved integration of multiple high dimensional independent datasets has led to potentially robust biomarkers for the detection of early HCC. The signature genes that we identified could be considered as "*evolutionarily conserved cross-species biomarkers for early HCC with genomic copy number alterations*". Further studies are needed to identify if any of the potential biomarkers identified in this study can be readily and reproducibly detected in blood, urine or other bodily fluids. This could then form the basis of a useful diagnostic test for the detection of early HCC.

## Methods

### Animals

Male Sprague-Dawley rats were maintained at the King Fahad National Centre for Children's Cancer and Research Animal Facility. This facility is managed in accordance with AALAS regulations. Ten young adult (5 months) and ten old adult (21 months) animals were subjected to partial hepatectomy. Actual survival rates allowed for four animals in each group to be analyzed. The re-growth of one lobe of the liver was completed within one month, by which time the liver cells again became quiescent, which was confirmed by histological analysis. In parallel, separate animals were treated with diethylnitrosoamine (200 mg/kg), which was injected intraperitoneally to induce the formation of HCC. Once the early HCC formation became apparent within 2-4 weeks, the rats were sacrificed. In the partial hepatectomized animals, one unaffected lobe and the regenerated lobe of the liver were removed independently. In the carcinogenic treated animals the tumors were carefully dissected to avoid removing normal tissue. All tissues were snap-frozen and stored at -80°C until required for RNA isolation. Small pieces of tissue were removed for formalin fixation to be used for histological examination.

### Human Subjects

Twenty blood samples were collected for this study (10 early HCC and 10 from healthy controls). Histopathological classification of HCC and clinical staging of early HCC were performed according to International Working Party [90] as previously described [8]. Patients diagnosed with the early HCC and healthy controls were recruited under an institutional review board-approved project (RAC# 2060040); all subjects provided written, informed consent before entry in the study. A total of 4 ml (in two separate PaxGene tubes) of whole blood samples were collected for each individual according to manufacturer's guidelines (QIAGEN Inc., Valencia, CA, USA). The total RNA isolation was performed using PreAnalytiX - PAXgene Blood RNA System (QIAGEN Inc.) by strictly following the manual and protocols provided with the kit-system.

### Microarray Hybridization

Total RNA was isolated according to standard protocols. Quality Control of RNA was done using Bioanalyzer 2100 RNA 6000 NanoAssay and RNA above RIN = 8 was included to the study (Agilent Technologies, Santa Clara, USA). Rat Genome Survey Microarray (Applied Biosystems, Foster City, CA, USA) was utilized for microarray studies. cDNA synthesis, cRNA and labeling, chemiluminescence detection, image acquisition and analysis were performed following the manufacturer's protocols, guidelines and recommendations.

### Microarray Data Analysis

Images were auto-gridded and the chemiluminescent signals were quantified, then background subtracted using the Applied Biosystems 1700 Chemiluminescent Microarray Analyzer software v 1.1. For transcriptome analysis, detection thresholds were used following the manufacturer's recommendations. Detection threshold was set as S/N > 3 and quality flag < 5000. The microarray data were analyzed from 24 samples (2 samples were excluded for quality reasons). The open source R software package http://www.r-project.org and tools from the BioConductor project were used for normalization and determination of differentially expressed genes [91]. Two-factor Analysis of Variance (ANOVA) was performed to include both "treatment" (HCC, regenerated and normal, which will be referred to as treatment in the remainder of the manuscript), as well as age (old and young) factor together with feature selection algorithm (also known as template matching(TMA)) [28] to look for treatment as well as age specific variation. Significantly modulated genes specific to HCC were defined as those with ANOVA (treatment) p- value < 0.01, and TMA p-value < 0.01. Additionally, samples were stratified as young and old cohorts, and HCC specific genes identified using one-way ANOVA in each age group separately. When comparing HCC group with regenerated and normal controls to identify the differentially expressed genes specific to the HCC, we used a combination of three criteria. We considered genes that are "present" in at least half of the samples in either group. HCC specific genes were defined as those with in absolute fold change > 1.8 and p-value < 0.02. These observations are consistent with the study of Guo *et. al*., in which gene lists ranked by fold change and filtered with non-stringent statistically significant tests were more reproducible across platforms than those generated through other analytical procedures [43]. A validation datasets were generated from three independent human HCC studies by Chiang *et. al*. [33] (GSE9843), which was composed of 91 HCV-related HCC tumor samples, of which 65 were very early or early stage disease (we used in our re-analysis only very early and early HCC datasets) and Boyault *et. al*. [34] (E-TABM-36) which consisted of 57 human HCCs and five samples of pooled non-tumorus tissues, and from Liao *et. al*. [35] (GSE 6222) consisting of various stages of HCC (we used expression data for only the early stage of the disease). Furthermore, we compared our results with the results from two independent studies [19,38] with the DEN-induced HCC in rats. The raw data was analyzed by using dChip[92] and open source R/Bioconductor packages. The dChip outlier detection algorithm was used to identify outlier arrays, and probes "present" in at least 50% of the samples in either group were filtered. The data was normalized by the GC Robust Multi-array Average (GC-RMA) algorithm [93,94]. Unpaired t-tests were performed to determine significant differences in gene expression levels between patients and normal controls. The Hierarchical clustering using Pearson's correlation with average linkage clustering was performed by MeV 4.0 [95].

Information about genes participating in known biological process and pathways were derived by using DAVID Bioinformatics Resources[96], Expression Analysis Systematic Explorer (EASE)[97], and PANTHER (Protein ANalysis THrough Evolutionary Relationships) Classification Systems [98]. For each molecular function, biological process or pathway term, PANTHER calculates the number of genes identified in that category in both a list of differentially regulated genes and a reference list containing all the probe sets present on the chip and compares these results using the binomial test to determine if there are more genes than expected in the differentially regulated list [99]. Over-representation is defined by p < 0.05. Statistical analyses were performed with the MATLAB software packages (Mathworks, Natick, MA, USA), R and Bioconductor and PARTEK Genomics Suite (Partek Inc, St. Lois, MO, USA).

### Functional Pathway and Network Analysis

Functional pathway, gene ontology and network analyses were executed using Ingenuity Pathways Analysis (IPA) 6.3 (Ingenuity Systems, Mountain View, CA). The differentially expressed signature gene lists for hepatoma and regeneration in different age groups were mapped to its corresponding gene object in the Ingenuity pathway knowledge base. These so-called focus genes were then used as a starting point for generating biological networks. A score was assigned to each network in the dataset to estimate the relevance of the network to the uploaded gene list. This score reflects the negative logarithm of the P that indicates the likelihood of the focus genes in a network being found together due to random chance. Using a 99% confidence level, scores of *≥*2 were considered significant. Significances for biological functions or pathways in the signature genes for such functions or pathways compared with the ABI Rat Genome Survey Microarray as a reference set. A right-tailed Fisher's exact test was used to calculate a p-value determining the probability that the biological function (or pathway) assigned to that data set is explained by chance alone.

### Cross-Species Comparative and Integrative Genomic Analysis

Human early HCC datasets from two independent studies by Mas *et. al*. [27] using Affymetrix HG-U133A 2.0 array, and Wumbach *et. al*. [8] using Affymetrix HG-U133 Plus 2.0 were re-analyzed. The raw data were analyzed using R/Bioconductor packages and Partek Genomics Suite (Partek Inc.). The data were normalized by the GC Robust Multi-array Average (GC-RMA) algorithm. Unpaired t-tests were performed to determine significant differences in gene expression levels between patients and normal controls. The cross mapping of Applied Biosystems Rat Genome Survey microarray probes were mapped to human orthologs through "AB1700 rat annotation spreadsheet" designed by Applied Biosystems on the basis of sequence identity. The transcripts present on both platforms (AB1700 and Affymetrix) were identified using Resourcerer [100]. Genes within copy number altered regions based on three independent genome CNA studies of human HCC [7,9,10] were determined using NCBI MapViewer http://www.ncbi.nlm.nih.gov/mapview, and integrated those with the gene expression profiling data (Figure 1).

### Realtime RT-PCR Experiments

Confirmatory realtime RT-PCR experiments were performed using the ABI 7500 Sequence Detection System (Applied Biosystems). 50 ng total RNA procured from the same microarray study samples were transcribed into cDNA using a Sensicript Kit (QIAGEN Inc., Valencia, CA, USA) under the following conditions: 25°C for 10 min, 42°C for 2 hrs, and 70°C for 15 min in a total volume of 20 μl. Six differentially expressed rat genes (*Pbsn, Cdh13, Lum, Nid2, Dcn, Slc22a5*) and eight human genes (*GJA1, VIM, IGFBP3, COL1A1, SP100, MMP2, LGALS3BP, *and *DPP4) *were selected and primers designed using Primer3 software. For the human samples, blood total RNA was utilized. After primer optimization, realtime PCR experiments were performed with 6 μl cDNA using Quantitech SyBr Green Kit (QIAGEN), employing *GAPDH *as the endogenous control gene. All reactions were conducted in triplicates and the data was analyzed using the delta delta C_T _method [101,102].

## Competing interests

The authors declare that they have no competing interests.

## Authors' contributions

DC and NK conceived the research problem and designed the methodology. MAC developed the rat model. MG provided suggestions for the rat model. NK and AA carried out microarray experiments. DC collected and processed the data, performed statistical and bioinformatics analyses, and drafted the manuscript together with NK and BHP. JQ and MMS provided suggestions for method design and commented on the manuscript. MAC, PTO, BHP, and AAQ provided general interpretation of results. All authors read and approved the final manuscript.

## Supplementary Material

Additional file 1 **Selected HCC specific genes, conserved across both age groups (old and young), and significantly modulated with respect to regenerated and normal liver**. Click here for file

Additional file 2 **Comparison of expression profiles of HCC and regeneration within the same age group**. (A, D) Heatmap of significantly dysregulated genes due to different treatment types in young and old, respectively. (B, E) Hierarchical clustering of samples separated based on treatment type in young and old, respectively. The gene expression clustering distance between the HCC group and other two groups (regenerated and normal) was the greatest in both age groups (C, F) Principle component analysis (PCA) which contained almost 76% of the variance in the data matrix clearly separated samples based on the treatment type in young and old, respectively. Click here for file

Additional file 3 **Heatmap and gene interaction networks of HCC specific genes in the old age group**. (A) Venn diagram characterizing differential gene expression between and specific to different treatment types (the HCC, the regenerated and the normal). The number of HCC specific genes, 100, is circled in *black*. (B) Heatmap of HCC specific genes exclusively dysregulated (up/down regulated) in the HCC group only. (C-E) Functional network analysis of HCC specific genes. Top three scoring gene interaction networks (with highest relevance scores) are shown. Nodes represent genes, with their shape representing the functional class of the gene product, and edges indicate biological relationship between the nodes (*see legend *in Figure 2). (F) Top network functions associated with three networks shown. An IPA score of three indicates that there is 1/1000 (score = -log (p-value)) chance that the focus genes are assigned to a network randomly. Click here for file

Additional file 4 **The gene interaction network analysis early HCC signature genes that are conserved in rat early HCC and in either of multiple human early HCCs **(A, B) The top two scoring gene interaction networks of 154 cross-species conserved signature genes indicated the importance of NF-κB, RAS and JNK activation in early hepatoma formation. Nodes represent genes, with their shape representing the functional class of the gene product, and edges indicate biological relationship between the nodes (*see legend *in Figure 5). Click here for file

Additional file 5 **The unsupervised Principle Components Analysis (PCA) was performed using our 35-gene signature to cluster samples from independent validation dataset of Chiang *et. al***. Our signature gene list was sufficient to separate individuals in Chiang *et al*.'s study as either early HCC patients or normal controls. Click here for file
