# Supplementary material for: Integrative and comparative genomics analysis of early hepatocellular carcinoma differentiated from liver regeneration in young and old
Source: Mol Cancer. 2010 Jun 12;9:146. doi: 10.1186/1476-4598-9-146 (PMC2898705; doi:10.1186/1476-4598-9-146)
Supplement: Additional file 1 — Selected HCC specific genes, conserved across both age groups (old and young), and significantly modulated with respect to regenerated and normal liver. [file 1476-4598-9-146-S1.PDF]

Additional file 1. **Selected rat early HCC specific genes, conserved across both age groups (old and young), and significantly modulated with respect regenerated and normal.**

Gene Symbol	Gene Name	Celera Gene ID	FC DO vs NO	FC DO vs RO	FC DY vs NY	FC DY vs RY	Biological Process
**Pbsn**	probasin	rCG36423	29.6	33.0	67.1	13.6	Pheromone response|Transport;Sensory perception
**Agtpbp1**	ATP/GTP binding protein 1 (predicted)	rCG24159	3.8	2.4	2.2	2.6	Proteolysis;Signal transduction|Protein metabolism and modification
**Col1a1**	collagen, type 1, alpha 1	rCG33732	3.7	3.2	2.2	3.7	Cell structure|Cell adhesion;Cell structure and motility
**Prom1**	prominin 1	rCG35777	3.4	2.8	1.8	1.9	Other intracellular protein traffic|Intracellular protein traffic
**Calb3**	calbindin 3, (vitamin D-dependent calcium binding protein)	rCG49631	3.0	2.9	4.3	3.8	Biological process unclassified
**Lum**	lumican	rCG48866	2.9	5.3	5.3	6.7	Vision|Signal transduction|Cell communication|Cell adhesion-mediated signaling;Sensory perception
**Fbn1**	fibrillin 1	rCG27283	2.9	1.9	1.6	1.6	Signal transduction|Cell communication;Sensory perception|Mesoderm development|Vision;Developmental processes|Skeletal development
**Star**	steroidogenic acute regulatory protein	rCG43004.1	2.4	2.4	2.5	2.0	Other lipid, fatty acid and steroid metabolism;Developmental processes|Regulation of lipid, fatty acid and steroid metabolism;Lipid, fatty acid and steroid metabolism|Gametogenesis|Oogenesis|Lipid, fatty acid and steroid metabolism
**Ccna2**	cyclin A2	rCG41610	2.3	3.0	1.2	1.6	Cell cycle control;Oncogenesis|Gametogenesis|Spermatogenesis and motility;Cell cycle|Developmental processes
**P2ry13**	purinergic receptor P2Y, G-protein coupled, 13	rCG50055	2.1	2.2	2.7	1.9	Cell surface receptor mediated signal transduction|Signal transduction|G-protein mediated signaling;Neuronal activities
**Tnfsf13**	tumor necrosis factor (ligand) superfamily, member 13	rCG34899	2.0	1.6	2.0	1.7	immune response|positive regulation of isotype switching to IgA isotypes|regulation of immune response|signal transduction|positive regulation of cell proliferation|humoral defense mechanism (sensu Vertebrata)
**Gja1**	gap junction membrane channel protein alpha 1	rCG22127	1.9	1.9	1.8	1.7	Signal transduction
**Ankrd22**	ankyrin repeat domain 22 (predicted)	rCG47624	1.8	1.9	3.3	2.5	Biological process unclassified
**Srpx**	sushi-repeat-containing protein	rCG42920	1.8	2.6	1.8	2.1	Biological process unclassified
**Abi2**	abl-interactor 2	rCG22366.1	1.6	2.4	2.3	2.3	Cell motility|Cell cycle control;Cell structure and motility|Signal transduction;Cell cycle
**Igfbp3**	insulin-like growth factor binding protein 3	rCG24498	1.6	1.7	1.4	1.6	Signal transduction|Growth factor homeostasis|Extracellular matrix protein-mediated signaling;Homeostasis|Cell communication
**Pnoc**	prepronociceptin	rCG52154.1	1.6	3.6	2.2	3.3	Cell surface receptor mediated signal transduction|Signal transduction|G-protein mediated signaling;Neuronal activities|Other neuronal activity
**Ctse**	cathepsin E	rCG46116.1	1.6	3.5	2.9	3.1	Proteolysis;Immunity and defense|Protein metabolism and modification|MHCII-mediated immunity|T-cell mediated immunity
**Stat2**	signal transducer and activator of transcription 2	rCG42435	1.4	1.5	1.5	1.6	Nucleoside, nucleotide and nucleic acid metabolism|Intracellular signaling cascade|mRNA transcription regulation;Signal transduction|Interferon-mediated immunity|mRNA transcription|JAK-STAT cascade;Immunity and defense|Macrophage-mediated immunity;Immunity and defense
**RT1-Ba**	RT1 class II, locus Ba	rCG60724	1.4	1.5	2.4	2.3	MHCII-mediated immunity|Immunity and defense|T-cell mediated immunity
**Hnrpa2b1**	Heterogeneous nuclear	rCG52521	1.4	1.2	1.5	1.5	Nucleoside, nucleotide and nucleic acid metabolism|Pre-mRNA

	ribonucleoprotein A2/B1 (predicted)						processing|mRNA splicing
**Lsp1**	lymphocyte specific 1	rCG47545.1	1.4	1.6	1.5	1.4	Biological process unclassified
**Creb1**	cAMP responsive element binding protein 1	rCG22512	1.3	1.1	1.9	1.4	Nucleoside, nucleotide and nucleic acid metabolism|mRNA transcription|mRNA transcription regulation;Signal transduction;Neuronal activities;Developmental processes|Neurogenesis|Ectoderm development
**Carhsp1**	calcium regulated heat stable protein 1	rCG49839.1	-1.2	-1.4	-1.6	-1.5	Intracellular signaling cascade|Nucleoside, nucleotide and nucleic acid metabolism;Signal transduction|Calcium mediated signaling;Other metabolism
**P2rxl1**	purinergic receptor P2X-like 1, orphan receptor	rCG36557	-1.4	-1.4	-1.8	-2.0	Hearing;Blood circulation and gas exchange|Synaptic transmission|Ion transport|Nerve-nerve synaptic transmission;Sensory perception|Vision;Sensory perception|Cation transport;Neuronal activities|Regulation of vasoconstriction, dilation|Signal transduction;Transport
**Eif4ebp1**	eukaryotic translation initiation factor 4E binding protein 1	rCG42993	-1.5	-1.2	-1.4	-1.3	Translational regulation;Cell proliferation and differentiation;Oncogenesis|Tumor suppressor|Protein biosynthesis;Protein metabolism and modification|Protein metabolism and modification
**Pfkfb4**	6-phosphofructo-2-kinase/fructose-2,6-biphosphatase 4	rCG25080	-1.7	-1.3	-2.1	-1.6	Glycolysis;Carbohydrate metabolism|Monosaccharide metabolism|Carbohydrate metabolism
**Kcnip2**	Kv channel-interacting protein 2	rCG57780	-1.7	-1.5	-2.5	-2.5	Intracellular signaling cascade|Calcium mediated signaling|Signal transduction
**Olr1602**	olfactory receptor 1602 (predicted)	rCG20131	-1.8	-2.2	-1.2	-2.0	Cell surface receptor mediated signal transduction|Signal transduction|G-protein mediated signaling;Sensory perception|Olfaction|Chemosensory perception
**Olr838**	olfactory receptor 838 (predicted)	rCG55011	-1.8	-3.5	-1.2	-1.5	Cell surface receptor mediated signal transduction|Signal transduction|Olfaction|Chemosensory perception|G-protein mediated signaling;Sensory perception
**Slc22a5**	solute carrier family 22 (organic cation transporter), member 5	rCG35332	-1.8	-2.0	-1.4	-1.3	Transport|Small molecule transport;Transport|Ion transport|Cation transport;Transport|Extracellular transport and import
**Gak**	cyclin G associated kinase	rCG57247	-1.8	-1.2	-1.6	-1.5	Protein metabolism and modification|Protein modification|Other protein metabolism;Intracellular protein traffic|Protein phosphorylation;Protein metabolism and modification|Endocytosis
**Ela1**	elastase 1, pancreatic	rCG50853	-2.0	-2.6	-3.6	-4.0	Proteolysis|Other steroid metabolism;Protein metabolism and modification|Cholesterol metabolism;Lipid, fatty acid and steroid metabolism|Steroid metabolism|Lipid, fatty acid and steroid metabolism
**Apom**	apolipoprotein M	rCG38376	-2.0	-1.8	-1.5	-1.3	transport|lipid transport
**Pik3cb**	phosphatidylinositol 3-kinase, catalytic, beta polypeptide	rCG25555	-2.0	-1.6	-2.5	-2.4	Cell surface receptor mediated signal transduction|Receptor protein tyrosine kinase signaling pathway;Signal transduction|Inhibition of apoptosis|Lipid, fatty acid and steroid metabolism|Lipid metabolism;Signal transduction|Intracellular signaling cascade;Apoptosis
**Ech1**	enoyl coenzyme A hydratase 1, peroxisomal	rCG53633	-2.3	-1.4	-1.8	-1.7	Vitamin biosynthesis|Coenzyme metabolism;Coenzyme and prosthetic group metabolism|Carbohydrate metabolism;Lipid, fatty acid and steroid metabolism|Fatty acid metabolism|Fatty acid beta-oxidation;Coenzyme and prosthetic group metabolism|Vitamin metabolism
**Mgat5**	mannoside acetylglucosaminyltransferase 5	rCG59358	-2.8	-1.3	-2.4	-1.3	Protein metabolism and modification|Protein modification
**Csrp2bp**	cysteine and glycine-rich protein 2 binding protein (predicted)	rCG27524	-2.8	-2.3	-1.6	-1.3	Biological process unclassified
**Bai1**	brain-specific angiogenesis inhibitor 1 (predicted)	rCG59454	-3.2	-3.9	-1.8	-1.2	G-protein mediated signaling;Cell adhesion;Developmental processes|Cell surface receptor mediated signal transduction|Signal transduction|Mesoderm development|Angiogenesis
**Clcnkb**	chloride channel Kb	rCG30632	-4.0	-3.3	-2.1	-1.3	Transport|Ion transport|Anion transport

**Ap2b1**	adaptor-related protein complex 2, beta 1 subunit	rCG33026	-4.9	-4.6	-2.3	-2.0	General vesicle transport|Intracellular protein traffic
