# Supplementary material for: Integrative and comparative genomics analysis of early hepatocellular carcinoma differentiated from liver regeneration in young and old
Source: Mol Cancer. 2010 Jun 12;9:146. doi: 10.1186/1476-4598-9-146 (PMC2898705; doi:10.1186/1476-4598-9-146)

Additional file 2. Comparison of expression profile of HCC and regeneration within the same age group. (A, D) Heatmap of significantly dysregulated genes due to different treatment types in young and old, respectively. (B,E) Hierarchical clustering of samples separated based on treatment type in young and old, respectively. The gene expression clustering distance between the HCC group and other two groups (regenerated and normal) was the greatest in both age groups (C,F) Principle component analysis (PCA) which contained almost 76 % of the variance in the data matrix clearly separated samples based on the treatment type in young and old, respectively.

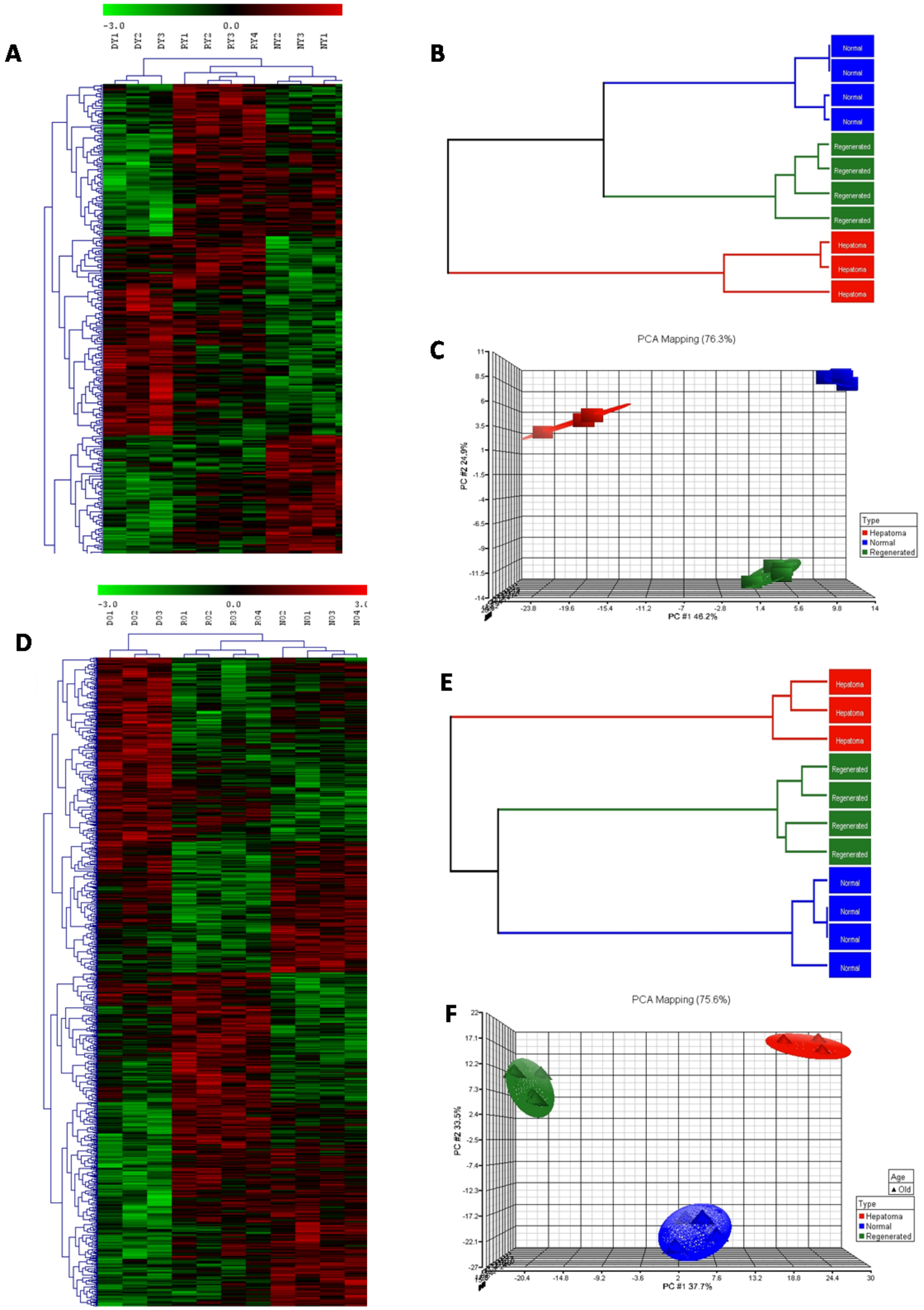

Supplement: Additional file 2 — Comparison of expression profiles of HCC and regeneration within the same age group. (A, D) Heatmap of significantly dysregulated genes due to different treatment types in young and old, respectively. (B, E) Hierarchical clustering of samples separated based on treatment type in young and old, respectively. The gene expression clustering distance between the HCC group and other two groups (regenerated and normal) was the greatest in both age groups (C, F) Principle component analysis (PCA) which contained almost 76% of the variance in the data matrix clearly separated samples based on the treatment type in young and old, respectively. [file 1476-4598-9-146-S2.PDF]
