# Supplementary material for: Integrative and comparative genomics analysis of early hepatocellular carcinoma differentiated from liver regeneration in young and old
Source: Mol Cancer. 2010 Jun 12;9:146. doi: 10.1186/1476-4598-9-146 (PMC2898705; doi:10.1186/1476-4598-9-146)
Supplement: Additional file 3 — Heatmap and gene interaction networks of HCC specific genes in the old age group. (A) Venn diagram characterizing differential gene expression between and specific to different treatment types (the HCC, the regenerated and the normal). The number of HCC specific genes, 100, is circled in black. (B) Heatmap of HCC specific genes exclusively dysregulated (up/down regulated) in the HCC group only. (C-E) Functional network analysis of HCC specific genes. Top three scoring gene interaction networks (with highest relevance scores) are shown. Nodes represent genes, with their shape representing the functional class of the gene product, and edges indicate biological relationship between the nodes (see legend in Figure 2). (F) Top network functions associated with three networks shown. An IPA score of three indicates that there is 1/1000 (score = -log (p-value)) chance that the focus genes are assigned to a network randomly. [file 1476-4598-9-146-S3.PDF]

Additional file 3. Heatmap and gene interaction networks of HCC specific genes in old age group. (A) Venn diagram characterizing differential gene expression between and specific to different treatment types (the HCC, the regenerated and the normal). The number of HCC specific genes, 100, is circled in *black*. (B) Heatmap of HCC specific genes exclusively dysregulated (up/down regulated) in the HCC group only. (C-E) Functional network analysis of HCC specific genes. Top three scoring gene interaction networks (with highest relevance scores) are shown. Nodes represent genes, with their shape representing the functional class of the gene product, and edges indicate biological relationship between the nodes (*see legend* in Figure 1). (F) Top network functions associated with three networks shown. An IPA score of three indicates that there is 1/1000 (score = -log (p-value)) chance that the focus genes are assigned to a network randomly.

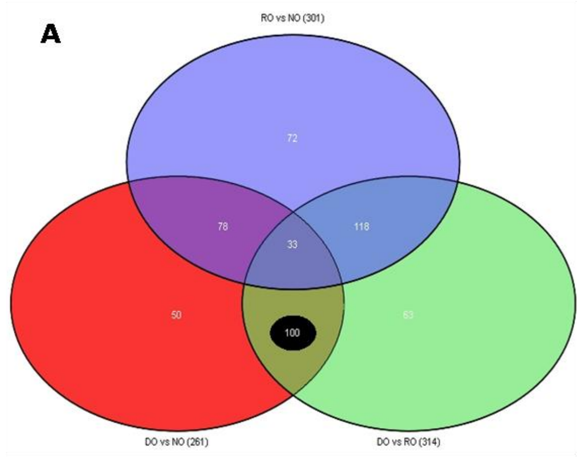

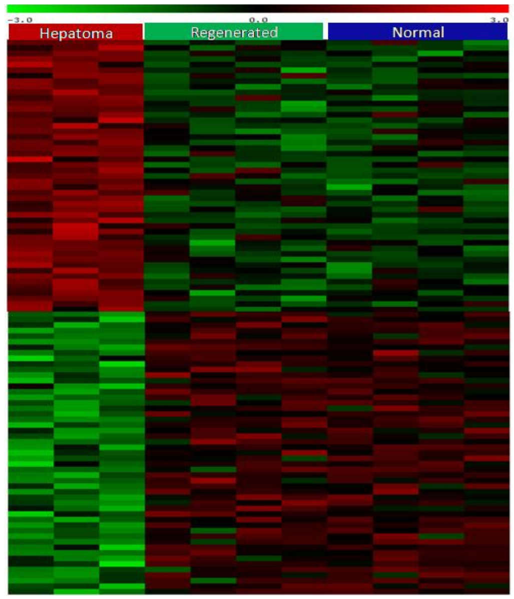

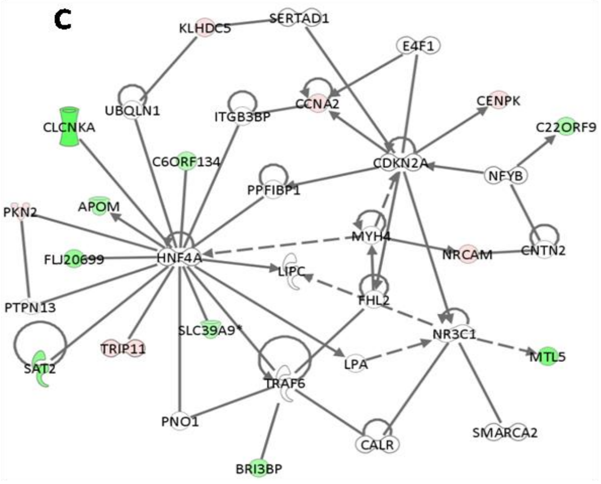

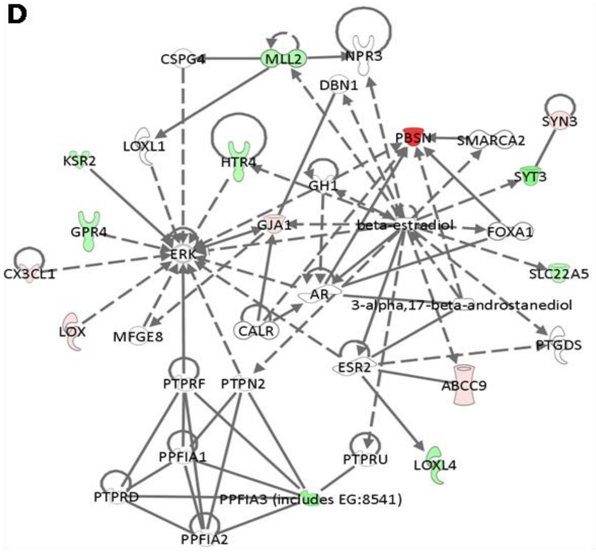

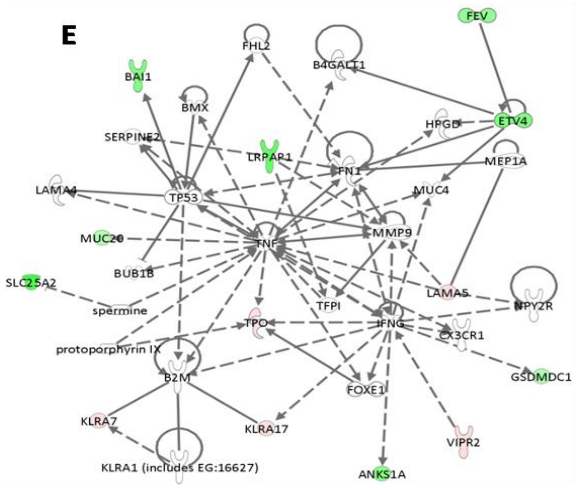

F

Top Network Functions	IPA Score
Cell Cycle, Cellular Development, Cellular Growth and Maintenance, and Cancer	32
Cancer, Cell-to-Cell Signaling and Interaction, Cellular Assembly and Organization, and Tissue Development	29
Cellular Movement, Cell Death, Embryonic Development, Cellular Growth And Proliferation, Cancer, and DNA Replication, Recombination And Repair	26
