# Supplementary material for: Integrative and comparative genomics analysis of early hepatocellular carcinoma differentiated from liver regeneration in young and old
Source: Mol Cancer. 2010 Jun 12;9:146. doi: 10.1186/1476-4598-9-146 (PMC2898705; doi:10.1186/1476-4598-9-146)

Additional file 4. The gene interaction network analysis early HCC signature genes that are conserved in rat early HCC and in either of multiple human early HCCs (A,B) The top two scoring gene interaction networks of 154 cross-species conserved signature genes indicated the importance of NFkB, RAS and JNK activation in the early hepatoma formation. Nodes represent genes, with their shape representing the functional class of the gene product, and edges indicate biological relationship between the nodes (*see legend* in Figure 5).

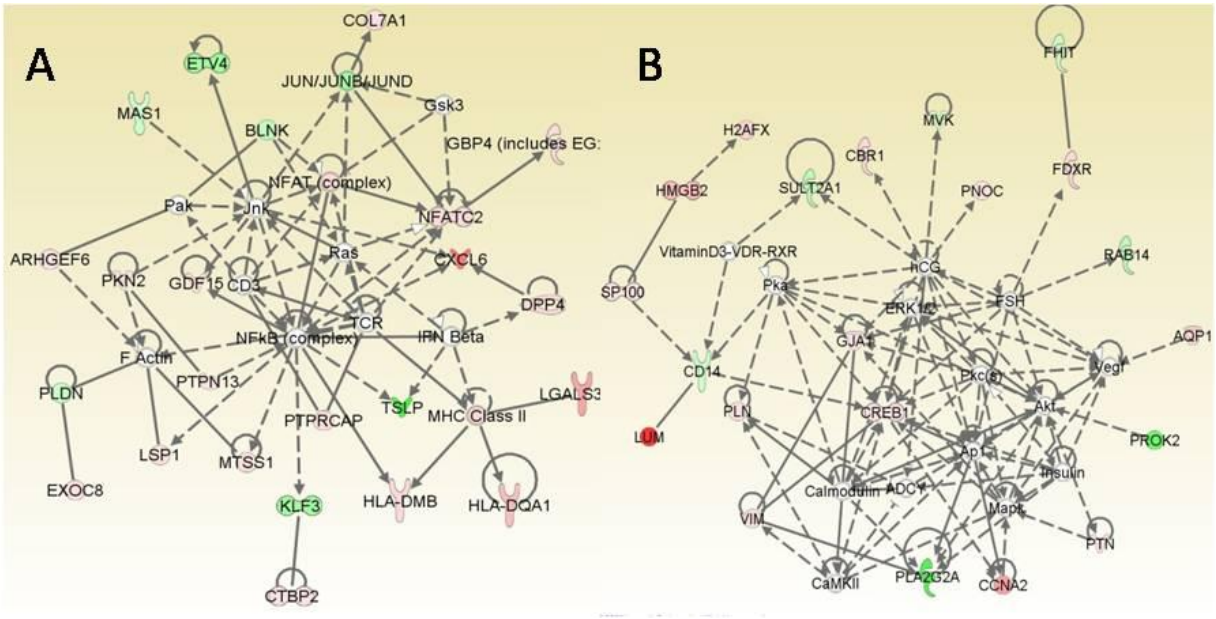

Supplement: Additional file 4 — The gene interaction network analysis early HCC signature genes that are conserved in rat early HCC and in either of multiple human early HCCs (A, B) The top two scoring gene interaction networks of 154 cross-species conserved signature genes indicated the importance of NF-κB, RAS and JNK activation in early hepatoma formation. Nodes represent genes, with their shape representing the functional class of the gene product, and edges indicate biological relationship between the nodes (see legend in Figure 5). [file 1476-4598-9-146-S4.PDF]
