# Supplementary figures and images for: Integrative and comparative genomics analysis of early hepatocellular carcinoma differentiated from liver regeneration in young and old
Source: Mol Cancer. 2010 Jun 12;9:146. doi: 10.1186/1476-4598-9-146 (PMC2898705; doi:10.1186/1476-4598-9-146)

PCA Mapping (46.8%)

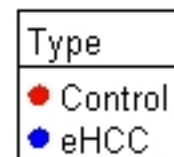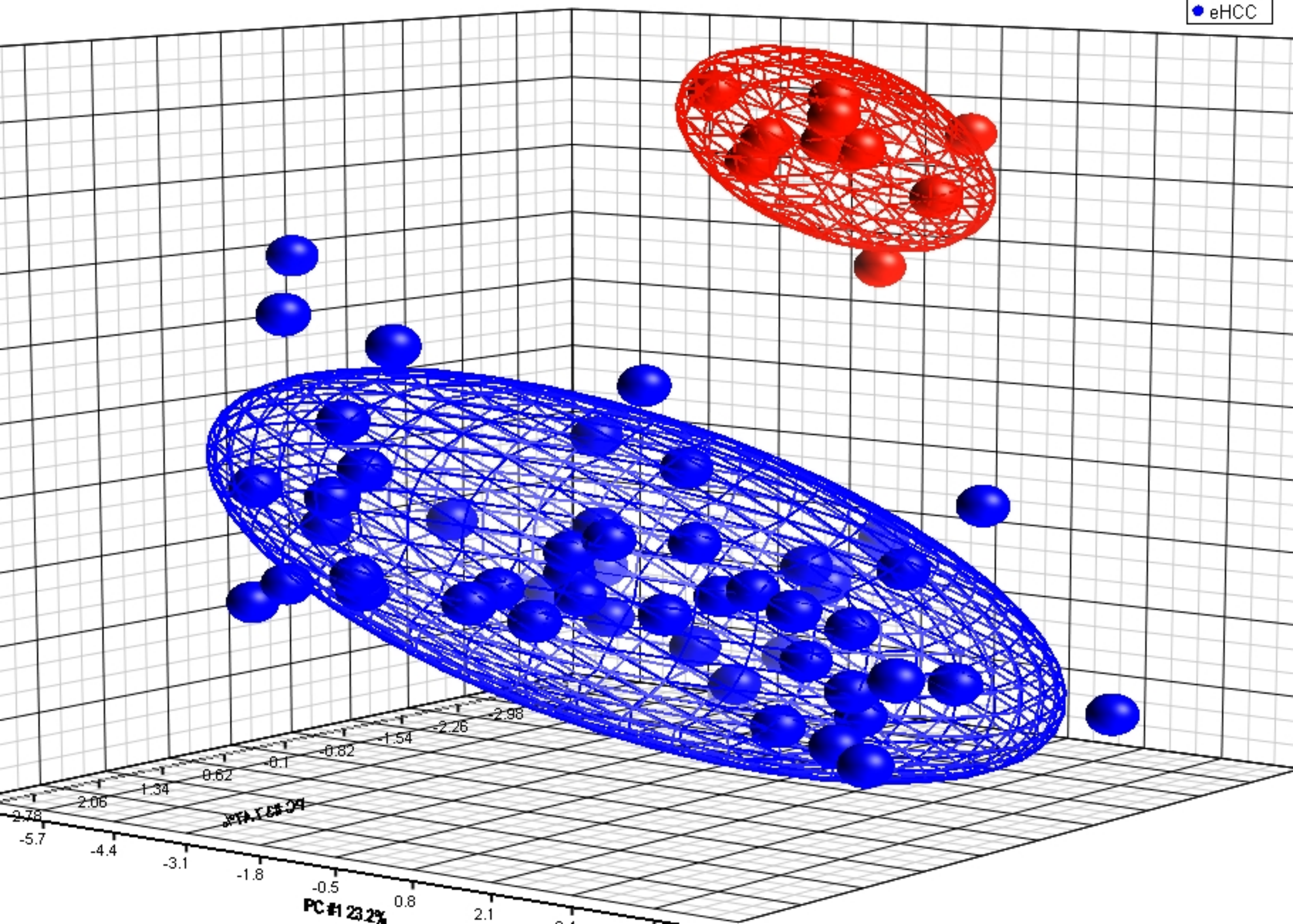

Supplement: Additional file 5 — The unsupervised Principle Components Analysis (PCA) was performed using our 35-gene signature to cluster samples from independent validation dataset of Chiang et. al. Our signature gene list was sufficient to separate individuals in Chiang et al.'s study as either early HCC patients or normal controls. [file 1476-4598-9-146-S5.PDF]
